# Supplementary material for: Tyrosine-targeted covalent inhibition of a tRNA synthetase aided by zinc ion
Source: Commun Biol. 2023 Jan 27;6:107. doi: 10.1038/s42003-023-04517-7 (PMC9880928; doi:10.1038/s42003-023-04517-7)
Supplement: Supplementary file 2 — Supplementary Information [file 42003_2023_4517_MOESM2_ESM.pdf]

Supplementary Information for

Tyrosine-targeted covalent inhibition of a tRNA synthetase aided by zinc ion

Hang Qiao[1], Mingyu Xia[1], Yiyuan Cheng[1], Jintong Zhou[1,2], Li Zheng[1,2], Wei Li[3], Jing Wang[1,2]*, Pengfei Fang[1,2]*

Correspondence to: jwang@sioc.ac.cn (J.W.); fangpengfei@sioc.ac.cn (P.F.)

This PDF file includes:

Supplementary Figures 1 to 10
Supplementary Tables 1 to 5

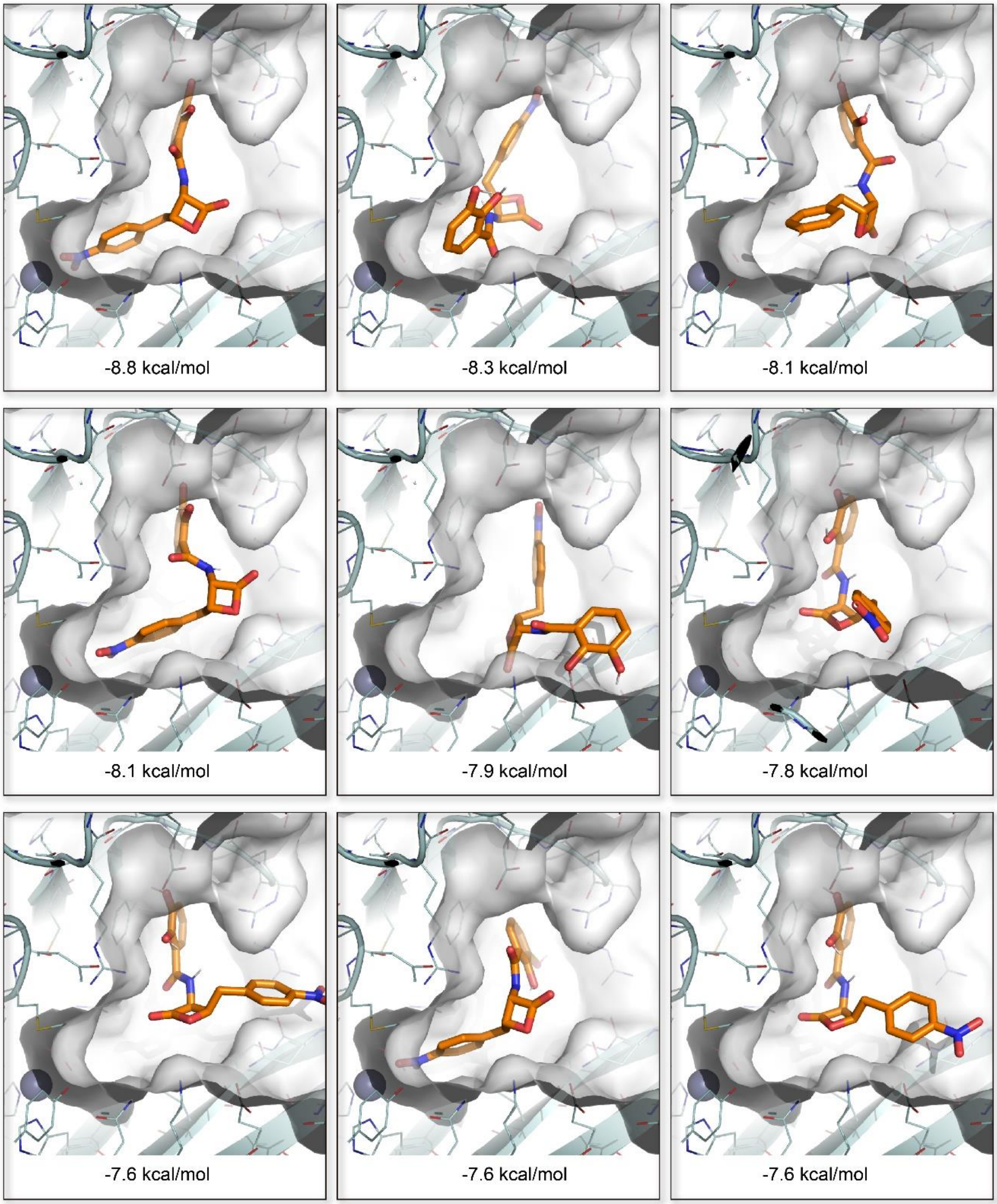

Supplementary Figure 1.

Molecular docking of OB in the active pocket of ThrRS. Nine highest scoring poses of OB are shown as orange sticks. The catalytic site of *E. coli* ThrRS is shown as light cyan lines, cartoons, and a surface. The conserved Zn^{2+} ion in ThrRS active site is shown as blue spheres.

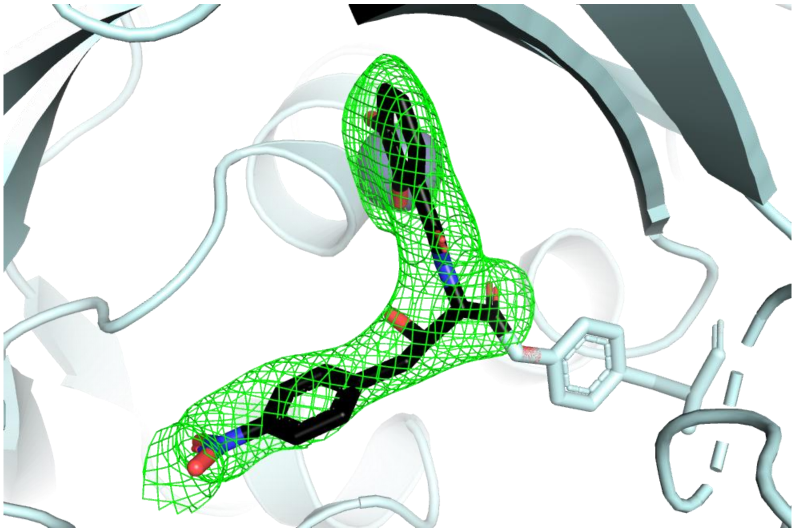

Supplementary Figure 2.

The Fo-Fc electron density is calculated with the OB-omitted structure model (made from the OB-ThrRS_WT structure), contoured at 3.0 σ, and shown as green meshes.

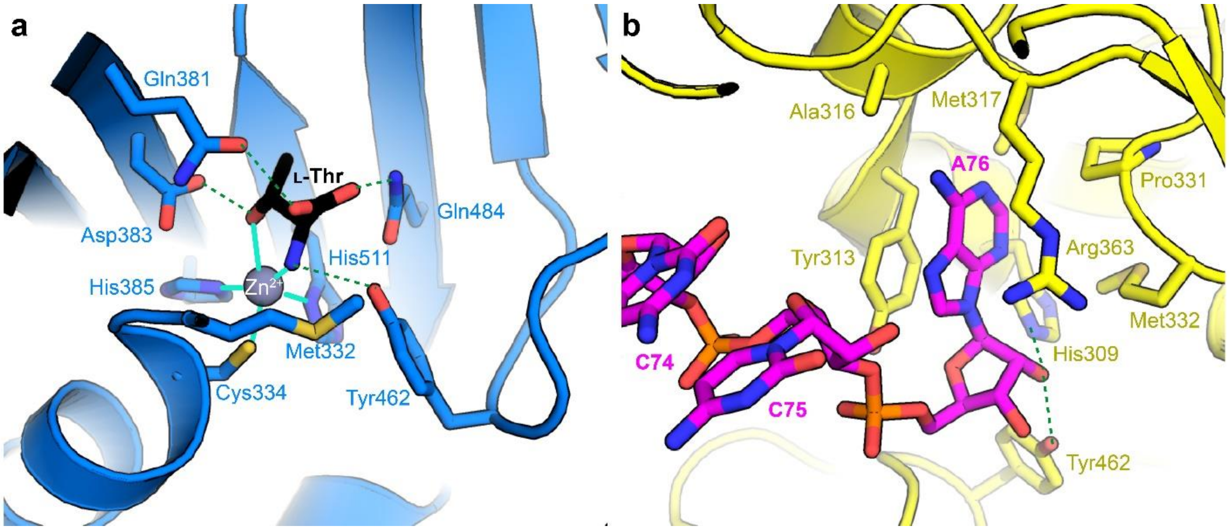

Supplementary Figure 3.

Molecular recognition of substrate L-threonine (L-Thr) and tRNA by ThrRS. (**a**) Close-up view of the L-Thr binding pocket of *E. coli* ThrRS–L-Thr structure (PDB: 1EVK). ThrRS protein is shown as blue cartoons. The residues interacting with L-Thr are shown as sticks. The substrate L-Thr is shown as black sticks. The co-bound Zn^{2+} ion is shown as a sphere. (**b**) Close-up view of tRNA pocket of *E. coli* ThrRS–$tRNA^{Thr}$ structure (PDB: 1QF6). ThrRS protein is shown as yellow cartoons. The residues interacting with tRNA A76 are shown as sticks. The 3' end of tRNA is shown as magenta sticks.

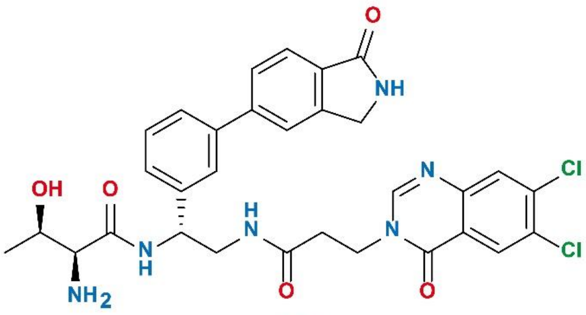

36J

Supplementary Figure 4.
Structure of compound 36j (PubChem CID: 163409105).

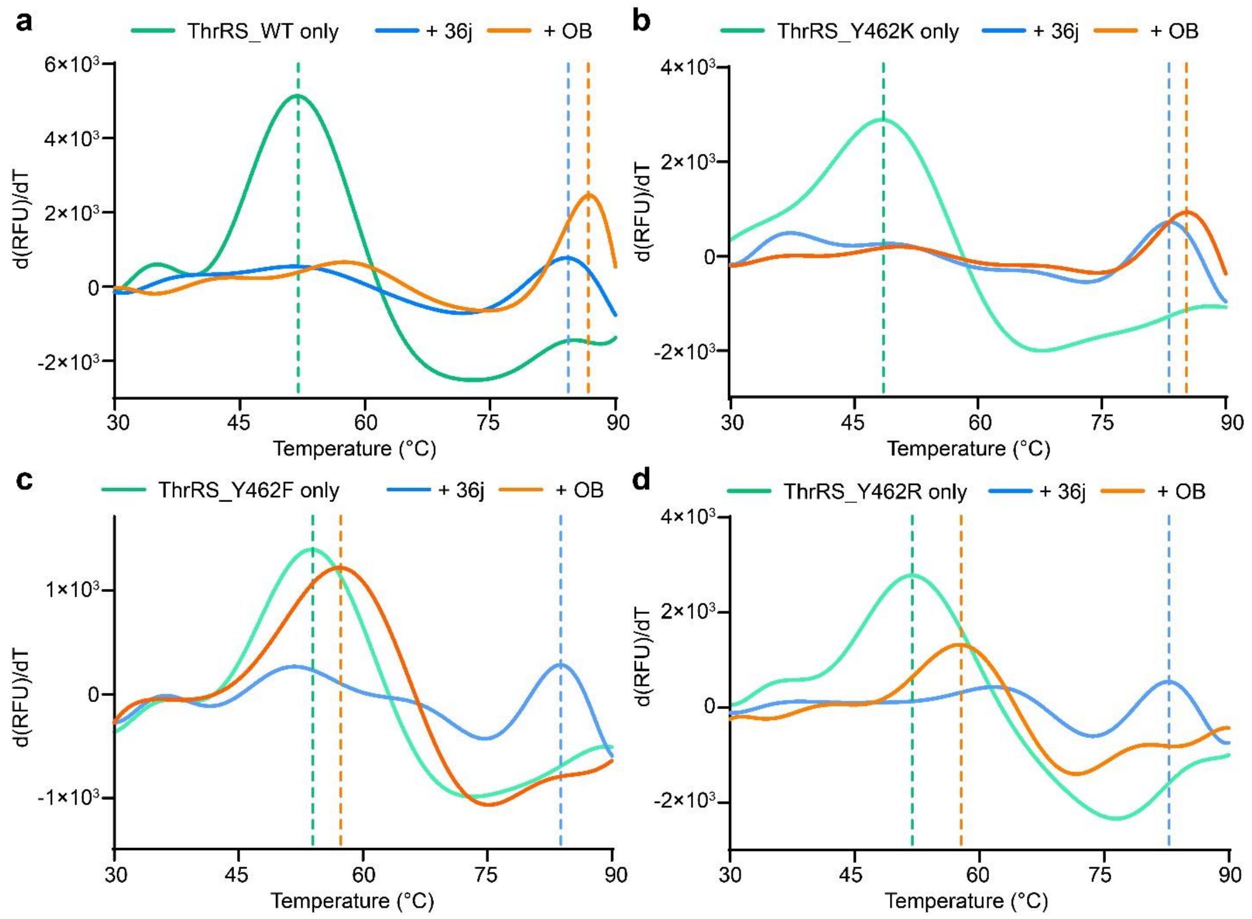

Supplementary Figure 5.

First derivatives of the melting curves for *E. coli* ThrRS_WT/Y462K/Y462F/Y462R in the presence or absence of inhibitors (OB or 36j). Data are representative of four independent assays. (**a**) ThrRS_WT. (**b**) ThrRS_Y462K. (**c**) ThrRS_Y462F. (**d**) ThrRS_Y462R.

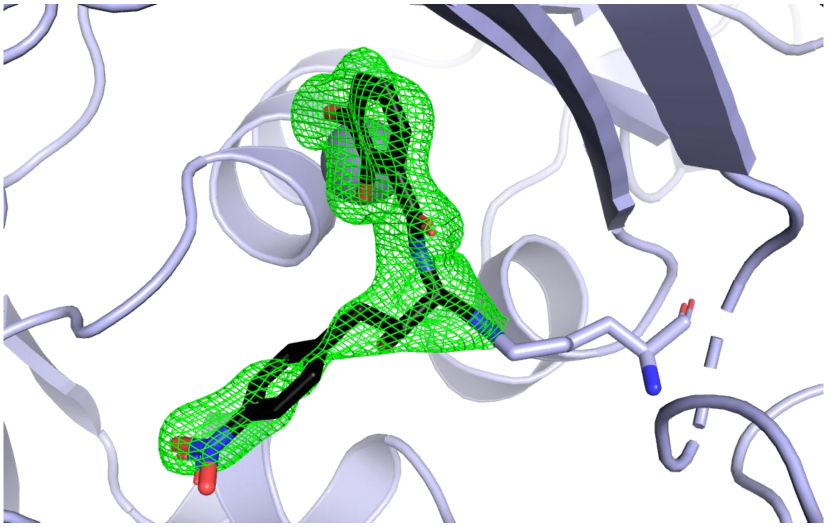

Supplementary Figure 6.

The Fo-Fc electron density is calculated with the OB-omitted structure model (made from the OB-ThrRS_Y462K structure which was obtained in the absence of ATP), contoured at 3.0 σ, and shown as green meshes.

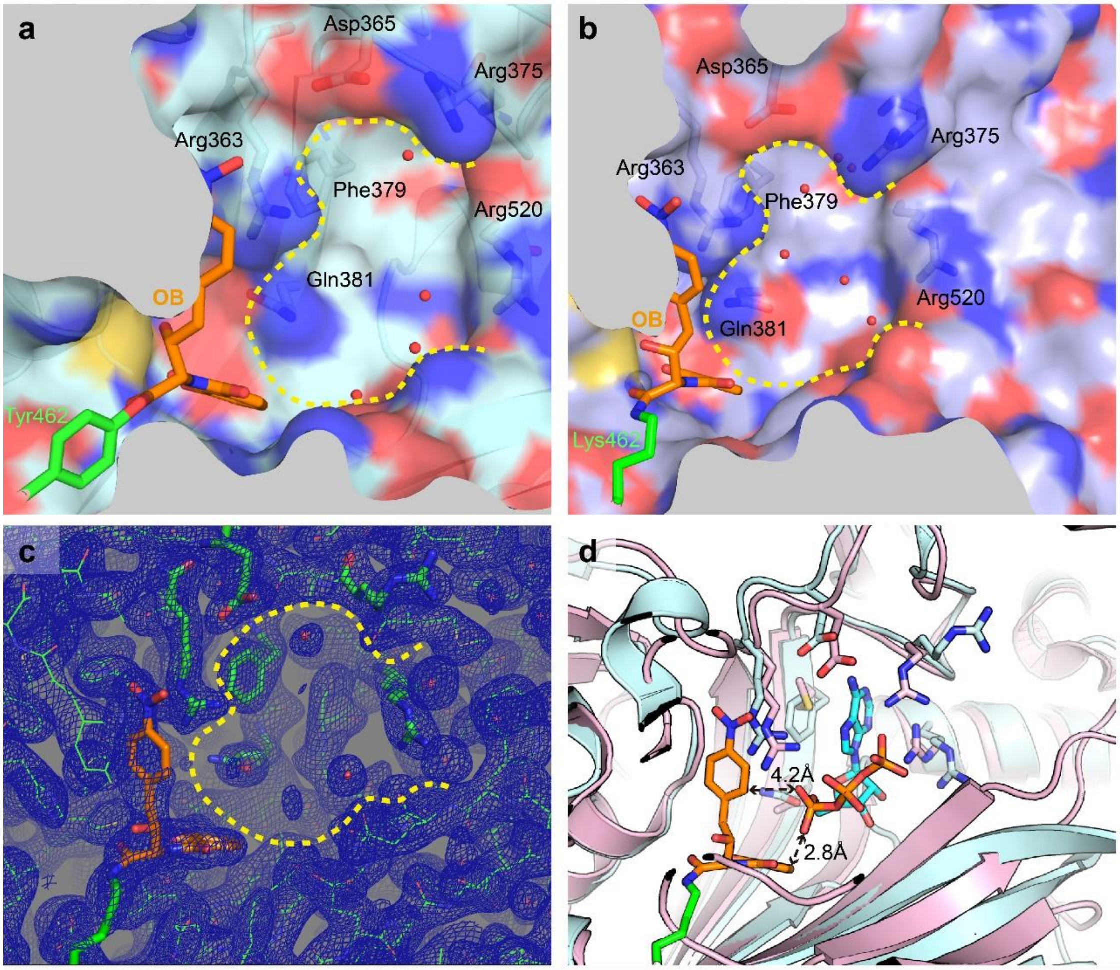

Supplementary Figure 7.

OB repels ATP binding by ThrRS. (**a**) Close-up view of ATP-binding pocket of ThrRS_WT–OB structure. The protein is shown as a transparent surface. The ATP binding site is indicated by yellow dotted line. OB is shown as orange sticks. The Tyr462 residue is shown as green sticks. (**b**) Close-up view of ATP-binding pocket of ThrRS_Y462K–OB which was crystallized in the absence of ATP. The protein is shown as a transparent surface. The ATP binding site is indicated by a yellow dotted line. OB is shown as orange sticks. The Lys462 residue is shown as green sticks. (**c**) Zoomed-in view of ATP-binding pocket of ThrRS_Y462K–OB which was crystallized in the presence of ATP. The 2Fo-Fc electron map (blue meshes contoured at 1.0 σ) is shown together with the structure model. The ATP-binding pocket is indicated by a yellow dotted line. OB is shown as orange sticks. The Lys462 residue is shown as green sticks. (**d**) Superimposition of the ThrRS_Y462K–OB structure (light cyan) onto *Staphylococcus aureus* ThrRS–ATP structure (light pink). OB and ATP are shown as sticks. The distances between the alpha phosphate of ATP and the two benzene rings of OB are 2.8 Å and 4.2 Å.

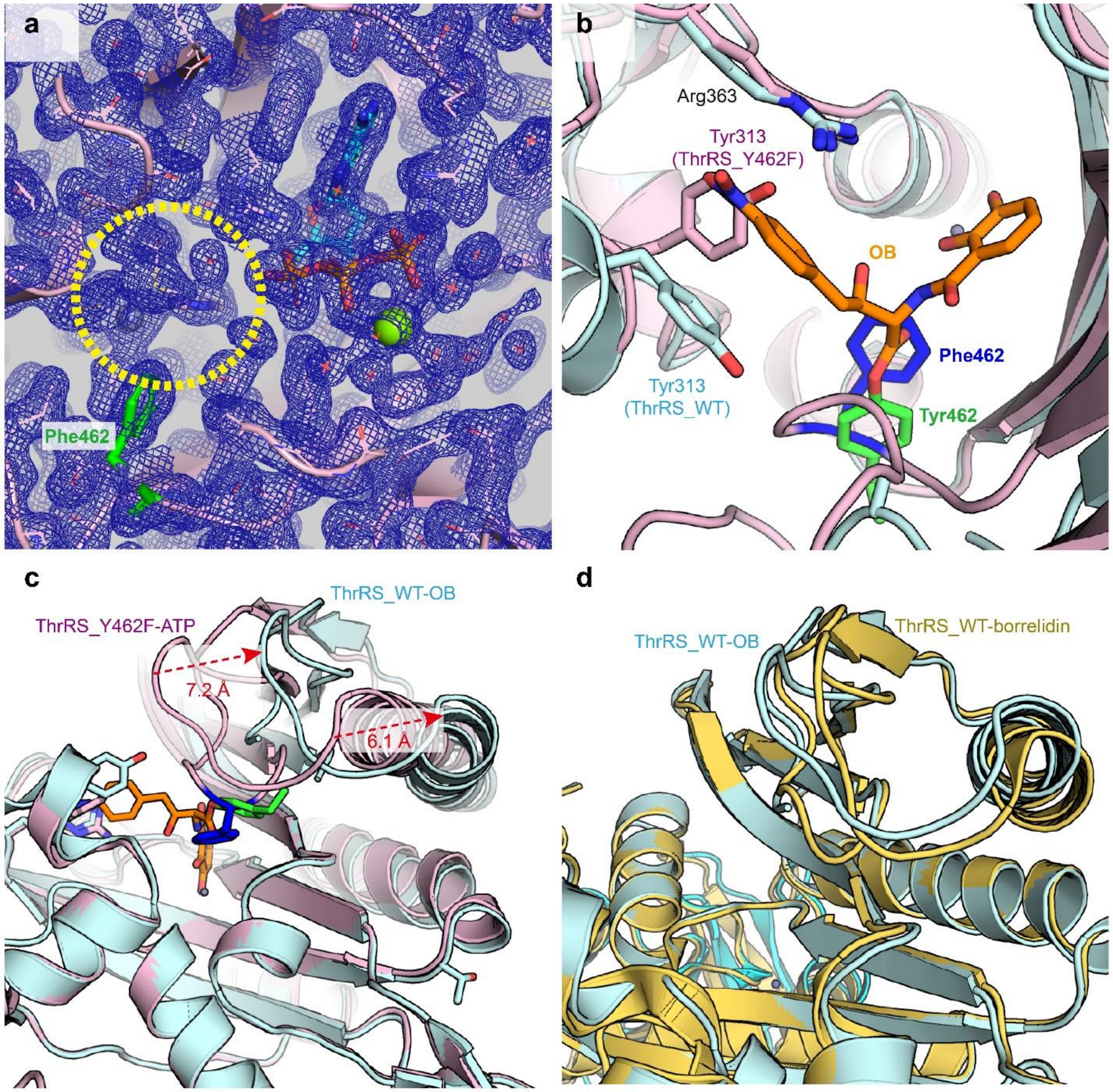

Supplementary Figure 8.

ATP repels OB binding by ThrRS_Y462F mutant. (**a**) Zoomed-in view of OB-binding site of ThrRS_Y462F–ATP structure which was crystallized in the presence of OB. The 2Fo-Fc electron map (blue meshes contoured at 1.0 σ) is shown together with the structure model. The OB-binding site is indicated by a yellow dotted line. The Phe462 residue is shown as green sticks. (**b**) Superimposition of the ThrRS_Y462F–ATP structure (light pink) onto ThrRS_WT–OB complex structure (light cyan) showing that Tyr313 in the ThrRS_Y462F–ATP clashes with the OB in the ThrRS_WT–OB structure. (**c**) Superimposition of the ThrRS_Y462F–ATP structure (light pink) onto ThrRS_WT–OB complex structure (light cyan) showing that OB induces a large conformational change to the the outer side of the active site cleft. (**d**) Superimposition of the ThrRS_WT–OB structure (light cyan) onto *E. coli* ThrRS–borrelidin complex structure (yellow, PDB: 4P3O).

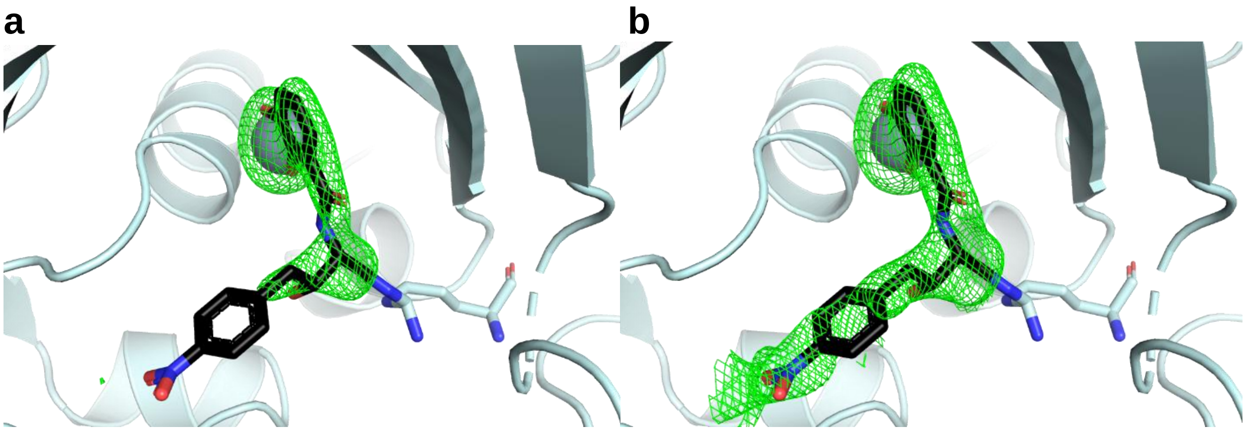

Supplementary Figure 9.

The Fo-Fc electron density is calculated with the OB-omitted structure model (made from the OB-ThrRS_Y462R structure), contoured at 3.0 σ (a) and 2.0 σ (b), and shown as green meshes. The nitrobenzene part has a weaker electron density than the other parts probably because the longer side chain of the arginine mutant leads to a higher temperature factor of this part.

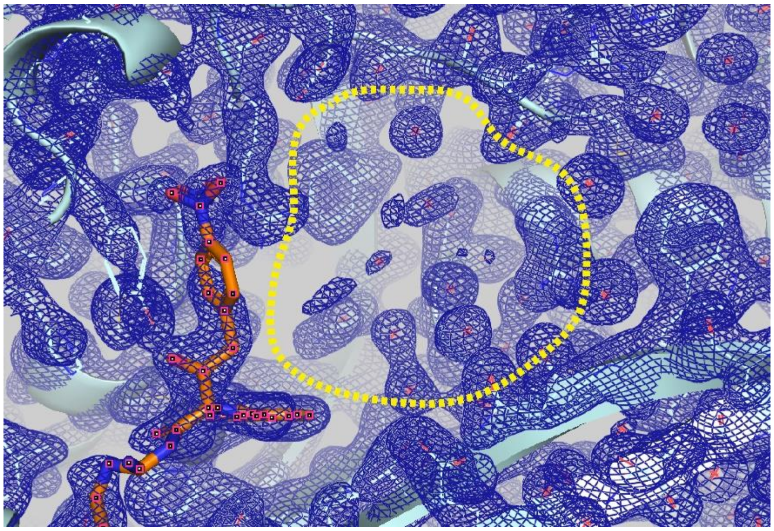

Supplementary Figure 10.

OB repels ATP binding by ThrRS_Y462R. Zoomed-in view of ATP-binding pocket of ThrRS_Y462R–OB which was crystallized in the presence of ATP. The 2Fo-Fc electron map (blue meshes contoured at 1.0 σ) is shown together with the structure model. The ATP-binding pocket is indicated by a yellow dotted line. OB is shown as orange sticks.

Supplementary Table 1.

Data collection and refinement statistics of the complex structure of *E. coli* ThrRS wild type protein and obafluorin (OB)

	ThrRS_WT-OB
PDB code	8H98
**Data collection**	
Space group	$P2_122_1$
Cell dimensions	
a, *b*, *c* (Å)	95.75, 105.91, 134.70
α, β, γ (°)	90.00, 90.00, 90.00
Resolution (Å)	50.00-2.50 (2.59-2.50)*
R_{sym} or R_{merge} (%)	8.0 (103.2)
I / s*I*	8.4 (1.5)
Completeness (%)	97.0 (98.5)
Redundancy	4.4 (4.5)
**Refinement**	
R_{work} / R_{free} (%)	19.1/23.0
No. atoms	
Protein	6487
Ligand	52
Metal	2
Solvent	274
B-factors (Å^2)	
Protein	48.31
Ligand	48.07
Metal	36.13
Solvent	45.44
R.m.s. deviations	
Bond lengths (Å)	0.009
Bond angles (°)	1.033
Ramachandran plot	
Most favored [%]	98.61
Additional allowed [%]	1.39

*Values in parentheses are for highest-resolution shell.

Supplementary Table 2.

Data collection and refinement statistics of the complex structure of *E. coli* ThrRS Y462K mutant and OB

	ThrRS_Y462K-OB[#]
PDB code	8H9A
**Data collection**	
Space group	$P3_221$
Cell dimensions	
a, *b*, *c* (Å)	91.62, 91.62, 121.54
α, β, γ (°)	90.00, 90.00, 120.00
Resolution (Å)	50.00-1.89 (1.94-1.89)*
R_{sym} or R_{merge} (%)	9.6 (60.6)
I / s*I*	10.1 (2.8)
Completeness (%)	98.1 (85.2)
Redundancy	8.5 (3.9)
**Refinement**	
R_{work} / R_{free} (%)	18.1/23.3
No. atoms	
Protein	3213
Ligand	26
Metal	1
Solvent	305
B-factors (Å^2)	
Protein	28.45
Ligand	36.69
Metal	18.10
Solvent	33.94
R.m.s. deviations	
Bond lengths (Å)	0.013
Bond angles (°)	1.295
Ramachandran plot	
Most favored [%]	98.98
Additional allowed [%]	1.02

*Values in parentheses are for highest-resolution shell.

[#]The protein was co-crystallized with OB in the absence of ATP.

Supplementary Table 3.

Data collection and refinement statistics of the complex structure of *E. coli* ThrRS Y462K mutant and OB

	ThrRS_Y462K-OB[#]
PDB code	8H9B
**Data collection**	
Space group	$P3_221$
Cell dimensions	
a, *b*, *c* (Å)	92.63, 92.63, 121.07
α, β, γ (°)	90.00, 90.00, 120.00
Resolution (Å)	50.00-2.20 (2.27-2.20)*
R_{sym} or R_{merge} (%)	6.5 (107.2)
I / s*I*	11.5 (1.1)
Completeness (%)	99.9 (100.0)
Redundancy	5.2 (4.4)
**Refinement**	
R_{work} / R_{free} (%)	19.7/23.2
No. atoms	
Protein	3219
Ligand	26
Metal	1
Solvent	97
B-factors (Å^2)	
Protein	38.86
Ligand	38.17
Metal	30.96
Solvent	34.31
R.m.s. deviations	
Bond lengths (Å)	0.008
Bond angles (°)	1.028
Ramachandran plot	
Most favored [%]	98.73
Additional allowed [%]	1.27

*Values in parentheses are for highest-resolution shell.

[#]The protein was co-crystallized with OB in the presence of ATP.

Supplementary Table 4.

Data collection and refinement statistics of the complex structure of *E. coli* ThrRS Y462F mutant and ATP

	ThrRS_Y462F-ATP[#]
PDB code	8H99
**Data collection**	
Space group	$P2_12_12_1$
Cell dimensions	
a, *b*, *c* (Å)	86.73, 109.99, 114.70
α, β, γ (°)	90.00, 90.00, 90.00
Resolution (Å)	50.00-1.94 (1.99-1.94)*
R_{sym} or R_{merge} (%)	10.0 (90.8)
I / s*I*	8.9 (1.7)
Completeness (%)	100.0 (99.9)
Redundancy	6.2 (3.8)
**Refinement**	
R_{work} / R_{free} (%)	17.7/20.2
No. atoms	
Protein	6468
Ligand	62
Metal	6
Solvent	708
B-factors (Å^2)	
Protein	25.42
Ligand	18.15
Metal	22.77
Solvent	33.32
R.m.s. deviations	
Bond lengths (Å)	0.009
Bond angles (°)	0.987
Ramachandran plot	
Most favored [%]	98.87
Additional allowed [%]	1.13

*Values in parentheses are for highest-resolution shell.

[#]The protein was co-crystallized with ATP in the presence of OB.

Supplementary Table 5.

Data collection and refinement statistics of the complex structure of *E. coli* ThrRS Y462R mutant and OB

	ThrRS_Y462R-OB[#]
PDB code	8H9C
**Data collection**	
Space group	$P3_221$
Cell dimensions	
a, *b*, *c* (Å)	92.18, 92.18, 120.68
α, β, γ (°)	90.00, 90.00, 120.00
Resolution (Å)	50.00-2.14 (2.20-2.14)*
R_{sym} or R_{merge} (%)	8.5 (77.8)
I / s*I*	10.0 (1.8)
Completeness (%)	100.0 (100.0)
Redundancy	9.9 (7.9)
**Refinement**	
R_{work} / R_{free} (%)	22.6/27.6
No. atoms	
Protein	3239
Ligand	26
Metal	1
Solvent	189
B-factors (Å^2)	
Protein	18.77
Ligand	30.67
Metal	24.05
Solvent	16.17
R.m.s. deviations	
Bond lengths (Å)	0.002
Bond angles (°)	0.547
Ramachandran plot	
Most favored [%]	98.98
Additional allowed [%]	1.02

*Values in parentheses are for highest-resolution shell.

[#]The protein was co-crystallized with OB in the presence of ATP.
