# Supplementary material for: Tyrosine-targeted covalent inhibition of a tRNA synthetase aided by zinc ion
Source: Commun Biol. 2023 Jan 27;6:107. doi: 10.1038/s42003-023-04517-7 (PMC9880928; doi:10.1038/s42003-023-04517-7)
Supplement: Supplementary file 3 — Description of Additional Supplementary Files [file 42003_2023_4517_MOESM3_ESM.pdf]

Description of Additional Supplementary Files

File name: Supplementary Data 1
Description: The source data behind Figure 4a.

File name: Supplementary Data 2
Description: The source data behind Figure 5a.

File name: Supplementary Data 3
Description: The source data behind Figure 5b.
